# Osseous Union of Teeth

**Published:** 1852-01

**Authors:** H. Scott


### OSSEOUS UNION OF TEETH—SUPERNUMERARY TOOTH.

LANCASTER, O., Aug. 18th, 1851.

DR. J. TAYLOR—DEAR SIR: Permit me to present to the cabinet of the Ohio College of Dental Surgery, through you, the inclosed specimen of the osseous union of two teeth. It is, as you will see, a perfect union of the teeth from end to end, and is perhaps the only one in the United States. I have heard of several specimens of partial union. This is the only one that I have seen in my sixteen years' practice.* I have furnished Dr. M. Z. Kreider, of our place, with drawings and specifications of it, which he wishes to send to Dr. Harris for the American Journal of Dental Science.

The patient from which it is obtained is Mary, daughter of Charles and Mary Sneider, Germans. She is six and a half years old. She was brought to my office to have one of these teeth (the central) extracted, on account of the permanent tooth having appeared above the gum behind. I at once saw the

---

*The specimen presented by Dr. Scott is very perfect, and we consider it a valuable addition to the College Museum, We have one other of a similar character, being also the union of two deciduous teeth, and a third specimen, the union of two of these teeth with the crown of a third, apparently the permanent central incisor, and what is most strange, it is attached to the crowns of the deciduous teeth, near their cutting edges. We have one specimen of osseous union of two permanent teeth, looking, however, more like a superior dens sapientia and supernumerary. This we judge from the appearance of the roots, leaving only a suture like appearance where they are united.—ED.

value of the specimen, and removed it, though not without great difficulty, on account of the extreme fears of the child.

It consists of the right inferior, central, and lateral incisors.

SUPERNUMERAY TOOTH.

Some ten days since, while removing the lime from the teeth of a Miss L., aged thirty years, I saw far back, toward the soft palate, near the median line of the palatine arch, a white point, which, upon touching, proved to be hard.* I spoke to the lady upon the subject. She told me that the doctor said "it was a piece of the bones forming the roof of the mouth, and that it must not be disturbed." Upon further examination I found it to be what it is, and upon my assuring her that no harm would come from removing it, she permitted me to do so. The apex of the fang was towards the dental arch, and the crown towards the vault of the palate, and near the center it lay opposite the dens sapientiæ, and was supernumerary to that tooth, as its shape proves. It seemed to have no alveolar cavity, but was somewhat imbedded in the palatine bone. Its distance from the dental circle makes it rather an interesting specimen, does it not?

Yours with respect, H. Scott.

---

Raymond, Miss., August 31, 1851.

Dr. Taylor—Dear Sir: I received yours some time ago, in which you remind me of my duty to assist in preparing the report for Dr. Drake, I regret very much that the tables of my

*This tooth had strayed very far from its right place, and shows what singular freaks nature sometimes makes. We have more frequently found these supernumerary teeth in the mouths of those whose teeth were unusually developed, often having enlargements on the crowns, and roots proportionally out of character, giving evidence that this operation of the economy is unduly taxed. We would here remark, in explanation to Dr. Scott, to whom we are indebted for these specimens and report of cases, that we had filed his letter for publication in the last Register, but it was mislaid and not found until overhauling our papers for the present No.—Ed.

